# ZmSPL12 Enhances Root Penetration and Elongation in Maize Under Compacted Soil Conditions by Responding to Ethylene Signaling

**DOI:** 10.3390/plants13243525

**Published:** 2024-12-17

**Authors:** Hua Xu, Zhigang Zheng, Lei Ma, Qingyun Zhang, Lian Jin, Ke Zhang, Junjie Zou, Hada Wuriyanghan, Miaoyun Xu

**Affiliations:** 1 Biotechnology Research Institute, Chinese Academy of Agricultural Sciences, Beijing 100081, China; 15756291121@163.com (H.X.); malei20520@163.com (L.M.); sdwfzqy1996@163.com (Q.Z.); jla13453556961@163.com (L.J.); zhangk4840@163.com (K.Z.); 2 Key Laboratory of Forage and Endemic Crop Biotechnology, Ministry of Education, School of Life Sciences, Inner Mongolia University, Hohhot 010070, China; 3 State Key Laboratory for Conservation and Utilization of Subtropical Agro-Bioresources, College of Life Sciences, South China Agricultural University, Guangzhou 510642, China; carlzzg@163.com; 4 National Nanfan Research Institute (Sanya), Chinese Academy of Agricultural Sciences, Sanya 572025, China; 5 Hainan Seed Industry Laboratory, Sanya 572025, China

**Keywords:** maize, root cap, transcription factor, compacted soil, ethylene

## Abstract

Soil compaction poses a significant challenge in modern agriculture, as it constrains root development and hinders crop growth. The increasing evidence indicated that various phytohormones collaborate in distinct root zones to regulate root growth in compacted soils. However, the study of root development in maize under such conditions has been relatively limited. Here, we identified that the *ZmSPL12* gene, belonging to the SPL transcription factor family, plays a crucial and positive role in regulating root development in the compacted soil. Specifically, the overexpression of *ZmSPL12* resulted in significantly less inhibition of root growth than the wild-type plants when subjected to soil compaction. Histological analysis revealed that the capacity for root growth in compacted soil is closely associated with the development of the root cap. Further exploration demonstrated that *ZmSPL12* modulates root growth through regulating ethylene signaling. Our findings underscored that *ZmSPL12* expression level is induced by soil compaction and then enhances root penetration by regulating root cap and development, thereby enabling roots to thrive better in the compacted soil environment.

## 1. Introduction

Maize (*Zea mays* L.) is one of the most widely cultivated crops globally, with widespread applications in feed, bioenergy, and food production [1]. Roots, as vital underground organs, serve as the bridge between the aboveground parts and the intricate soil environment. They facilitate water and nutrient uptake while providing mechanical support for stem growth [2]. The morphology and structure of roots directly influence nutrient and water absorption, thereby impacting plant growth [3]. However, studying root systems under field conditions is challenging due to the spatial heterogeneity of soil, necessitating considerable effort to characterize maize roots [4]. Despite these hurdles, scientists have recognized the critical role of the root system and consider it crucial for enhancing crop yields, with some focusing even more intently than during the “Green Revolution” [5].

Soil compaction poses a significant limitation to root growth. In modern agriculture, mechanical pressure and tillage practices often lead to soil compaction, hindering crop roots from penetrating hard soil layers and reducing crop yields by approximately 25%. When combined with drought, yield losses can escalate to 75% [6,7]. Maize varieties with robust root penetration capabilities have an advantage in accessing deeper water and nutrients [8]. Therefore, breeding maize varieties with enhanced root growth under compacted soils can effectively mitigate the adverse effects of mechanical impedance [9].

Phytohormonal changes under soil compaction are pivotal in affecting maize root growth. Roots perceive soil compaction through ethylene: compacted soils decrease air-filled pore spaces, reducing gas diffusion and leading to ethylene accumulation in root tissues, which subsequently inhibits root growth [10]. Ethylene, in conjunction with abscisic acid (ABA) and auxin, inhibits root elongation. Ethylene utilizes auxin and ABA as downstream signals to alter root cell elongation and radial expansion in rice, causing root tip swelling and reducing the roots’ ability to penetrate compacted soils [9].

EILs (ethylene-insensitive-like transcription factors), an ethylene response gene, plays a crucial role in this process. Research conducted on rice showed that soil compaction leads to an increase in the transcription levels of *OsEIL1* in roots. *OsEIL1* directly binds to the promoter of the ethylene-regulated gene *OsWOX11*, both in vitro and in vivo, thereby activating its expression. Consequently, *OsWOX11* facilitates the initiation and development of crown root primordia by modulating the expression levels of *OsRR2* and *OsCKX4* [11,12]. The transcriptional activation of YUC8/REIN7 and the expression of indole-3-pyruvate (IPA)-dependent auxin by *OsEIL1* are necessary conditions for ethylene to inhibit root elongation [13]. The content of ethylene induced by compaction upregulates the auxin biosynthesis gene *OsYUC8*, and *OsYUC8* mutants exhibit enhanced penetration capabilities in compacted soil [9]. Furthermore, rice mutants of ABA biosynthesis genes exhibited reduced radial expansion of the cortical cells in compacted soil. Studies in *Arabidopsis* have demonstrated that ethylene plays a positive role in regulated auxin biosynthesis at the tips of roots. In the presence of auxin, the ethylene precursor 1-aminocyclopropane-1-carboxylic acid (ACC) significantly enhances its ability to suppress the elongation of root cells. By upregulating auxin biosynthesis, ethylene amplifies its capacity to inhibit root cell expansion [14].

In maize, low oxygen conditions induce the production of ACC oxidase, leading to an increase in the ethylene precursor and subsequently elevated ethylene levels to cope with hypoxic conditions [15]. Studies have shown that the content of Multiseriate cortical sclerenchyma (MCS) is positively correlated with soil permeability, and maize genotypes possessing MCS exhibit significantly increased root depth in compacted soil [16]. The maize root cap plays a crucial role in the ethylene response, as root elongation is no longer inhibited by ethylene when the root cap is removed. This result indicates that the root cap is essential for regulating ethylene-induced root elongation in maize [17].

The SPL transcription factors (Squamosa Promoter-Binding Protein-Like, SPL), a type of plant-specific transcription factor family, play crucial roles in plant growth and development, signal transduction, and responses to environmental stresses [18]. In diverse plant species, numerous *SPL* genes are targeted by microRNA156 (miR156), indicating that the miR156/SPL regulatory module exhibits evolutionary conservation in regulating distinct developmental processes throughout the plant kingdom [19]. In *Arabidopsis thaliana*, the overexpression of *SPL12* can enhance inflorescence heat tolerance and seed yield [20]. In *Medicago sativa*, the miR156/SPL13 network contributes to improving the heat stress tolerance of alfalfa [21]. In *Tripogon loliiformis*, miR157d and miR156 enhance drought resistance by downregulating *SPL17*, *SPL18*, and *SPL19* through targeting these genes [22]. Moreover, *VpSBP16* can enhance the tolerance of species to drought stress by regulating the SOS (salt overly sensitive) and ROS (reactive oxygen species) signal cascades [23]. Hence, the miR156/SPL regulatory module exerts significant functions in multiple abiotic stresses.

In modern agriculture, soil compaction has intensified significantly due to factors including mechanical harvesting, soil trampling, and variations in soil moisture levels. This impact is particularly pronounced for maize, a crop that thrives in dryland conditions [24,25,26,27,28,29]. At present, numerous studies are exploring the relationship between soil compaction and plant growth, with particularly swift advancements in *Arabidopsis* and rice. However, the research on maize remains limited, primarily focusing on phenotype investigations, with few studies addressing gene regulatory networks under soil compaction conditions. Here, our study showed that *ZmSPL12* overexpression lines grew better in compacted soil conditions, and the ethylene-responsive genes were downregulated by *ZmSPL12* overexpression. Consequently, this study will give more insight into understanding the comprehensive regulatory network of the root growth of maize in compacted soils.

## 2. Results

### 2.1. The Overexpression Lines of ZmSPL12 Exhibit Insensitivity to Soil Hardness

ZmSPL12 (Zm00001eb233320) belongs to the SPL transcription factor family members. At the outset of our study, the *ZmSPL12* overexpression (OE) and knockout (KO) materials were generated to test their sensitivity to soil hardness. Our previous research has demonstrated that *ZmSPL12* negatively regulates *D1,* which encodes a GA 3-oxidase catalyzing the final step of bioactive GA synthesis, thereby influencing maize plant height and lodging resistance [30]. During field trials, the brace roots of wild-type hybrids failed to penetrate the soil properly, whereas the hybrids overexpressing *ZmSPL12* remained largely unaffected. The findings indicate that *ZmSPL12* could play a role in modulating the root’s ability to penetrate soil of varying hardness.

**Figure 1:**
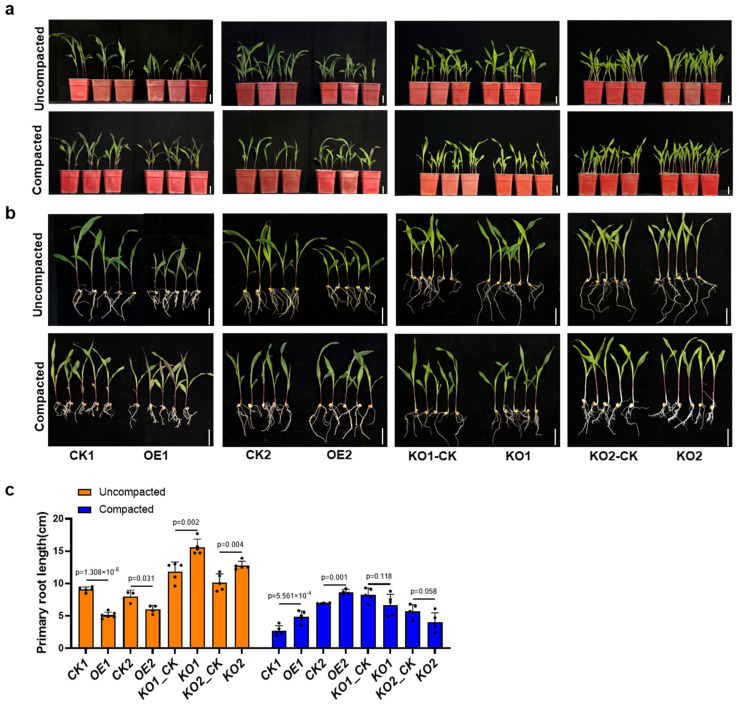
A comparison of the primary root phenotypes in *ZmSPL12* overexpression and KO plants under uncompacted and compacted soil conditions. (**a**,**b**) Phenotypes of the seedlings and primary roots of *ZmSPL12* overexpression lines, KO lines, and their corresponding controls under uncompacted (**top**) and compacted (**bottom**) soil conditions. Scale bars = 3 cm. (**c**) The primary root lengths of the *ZmSPL12* overexpression lines, KO lines, and their corresponding controls under both uncompacted and compacted soil conditions. The values are presented as means ± standard deviation (s.d.), with the individual data points indicated as black circles. The statistical significance was assessed using a two-tailed Student’s *t*-test.

To further investigate this phenomenon, we carried out a controlled soil compaction experiment in the laboratory. The control group comprised *ZmSPL12* overexpression and knockout lines, along with their corresponding wild-type counterparts, all grown under standard, loosened soil conditions. By contrast, the experimental group involved planting all maize seeds in compacted soil with a measured hardness of approximately 300 kPa. Normal soil was then layered over the seeds, followed by regular watering. After about three weeks of growth, the soil was carefully washed away, allowing us to capture phenotypic photographs and measure root lengths (Figure 1). Under loosened soil conditions, the primary root length of the *ZmSPL12* overexpression lines was significantly shorter than that of wild-type plants. Notably, an increase in soil hardness led to a significant suppression of root growth in wild-type plants. In contrast, the overexpression lines displayed a notably lesser inhibition of primary root growth compared to the wild-type (Figure 1a,b). These results revealed that *ZmSPL12* overexpression lines have longer roots than wild-type plants in compacted soil conditions. As for the *ZmSPL12* knockout lines, their primary root length was significantly longer than that of wild-type plants under normal growth conditions. When grown in compacted soil conditions, both knockout lines and wild-type plants exhibited inhibited primary root growth (Figure 1a,b). Interestingly, the degree of inhibition observed in the knockout lines was significantly higher than that observed in the wild-type plants (Figure 1c).

Subsequently, we endeavored to determine whether the crown root phenotype of *ZmSPL12* OE lines was consistent with that of the primary roots. Therefore, a crown root compaction experiment was conducted, prolonging the maize growth period to three months, similar to the seedling compaction experiment. Photographic analysis revealed that the crown roots of the OE lines had a somewhat less robust appearance than those of wild-type plants, despite no significant differences in length. Conversely, the crown roots of the KO lines exhibited greater length compared to those of the wild-type. With the increase in soil hardness, the crown root growth was suppressed in all lines. However, the impact of suppression on the OE lines was considerably less than that on the wild-type plants, whereas the impact on the KO lines was more intense than that of the wild-type plants (Supplementary Figure S1).

### 2.2. The Primary Roots of ZmSPL12-Overexpressing Lines Exhibited Diminished Sensitivity to Increased Soil Bulk Density

In previous experiments, we verified that the root elongation of the overexpressed lines is related to soil hardness. It has been reported that the increase in soil bulk density usually leads to a decrease in soil porosity, thereby increasing soil compaction. By changing soil bulk density, soil hardness can be altered [31,32]. Next, we conducted treatment experiments with different soil bulk densities (BDs).

**Figure 2:**
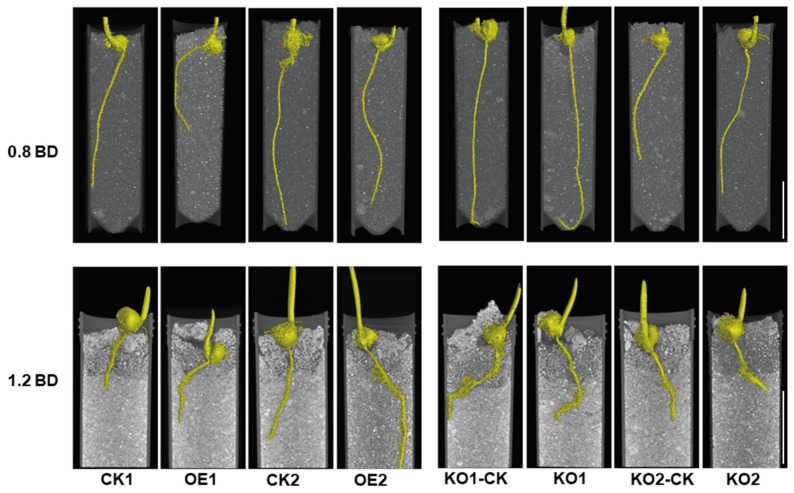
Overexpressing *ZmSPL1*2 enhances the penetration ability of maize roots into compacted soil. Under normal conditions, the primary root length of transgenic plants overexpressing *ZmSPL12* was notably shorter than that of non-transgenic controls. Conversely, the primary root length of the *ZmSPL12* KO lines was significantly longer than that of the corresponding controls. In compacted soil, a different trend was observed: the primary roots of the *ZmSPL12* OE lines exhibited substantial elongation compared to the controls. In contrast, the primary roots of the *ZmSPL12* KO lines showed significant shortening relative to the controls. The bulk density (BD) of the soil was varied, with uncompacted soil having a BD of 0.8 g cm^−3^ and compacted soil having a BD of 1.2 g cm^−3^, providing a controlled environment to assess root growth responses. Scale bars = 3 cm.

To further investigate the role of *ZmSPL12* under compacted soil, phenotypes of OE and KO lines and their wild types plants were verified under different soil bulk density conditions. For standard maize cultivation, a bulk density (BD) of 0.8 g cm⁻^3^ was considered to be normal density, and 1.2 g cm⁻^3^ was considered to be high density. Scanning and photographic analyses were performed to assess root phenotypes (Figure 2). Similar to those found in soil, the primary root length of the *ZmSPL12* OE lines grown in test tubes with normal BD was shorter than those of wild-type plants. However, the root growth of wild-type plants was significantly suppressed under high BD compared to the OE lines, indicating that the *ZmSPL12* OE lines have a higher penetration ability than the wild-type plants. Conversely, the primary root length of the KO lines was longer than that of wild-type plants under normal BD in the test tubes. Although both knockout and wild-type primary root growth were suppressed, the degrees of suppression were remarkably higher in the KO lines than in the wild-type plants (Figure 2).

It can be seen from the results that under low BD, the root elongation of the *ZmSPL12* overexpression line is lower than that of the wild type, but the increase in soil bulk density leads to an increase in soil hardness. The roots of the *ZmSPL12* overexpression line are not sensitive to soil hardness, so the inhibition is less than that of the wild type. The knockout lines exhibit higher root elongation at a low BD compared to the wild type. As soil bulk density increases, they become more sensitive to hardness due to the lack of the *ZmSPL12* gene, resulting in shorter primary roots compared to the wild type. Consistent with the results of the soil hardness test, the *ZmSPL12* overexpression line was not sensitive to high BD, and the *ZmSPL12* knockout line was sensitive to high BD. Therefore, we conclude that *ZmSPL12* is involved in regulating root development under compacted soil.

### 2.3. ZmSP12-Mediated Root Penetration Is Associated with Ethylene Signal

Previous reports have highlighted ethylene as a crucial factor influencing root growth under soil compaction conditions [10]. To confirm whether *ZmSPL12* functions in root penetration through regulating the ethylene signal, we conducted an ethylene response experiment by exposing the C01 line to ethylene for 24 h and collecting whole roots as controls. Subsequently, specific root regions including the root tip, elongation zone, root hair zone, maturation zone, and lateral root zone were isolated for qRT-PCR analysis. The results revealed that the changes in the expression levels of *ZmSPL12* in specific regions are different, indicating that *ZmSPL12* exhibits differential responses to ethylene across various root system regions (Supplementary Figure S2).

**Figure 3:**
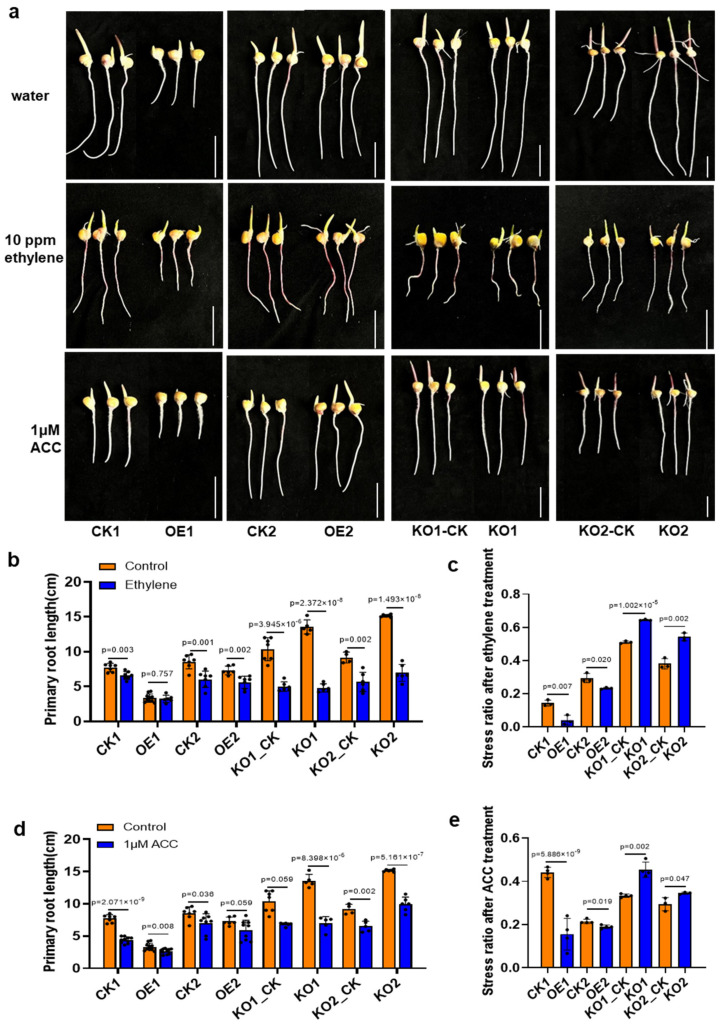
A comparison of the primary root phenotypes and growth parameters in the *ZmSPL12* overexpression and KO lines following treatment with ethylene or ACC. (**a**) The phenotypes of the primary roots in the *ZmSPL12* OE and KO lines, along with their corresponding controls, are depicted under water, ethylene, and ACC treatments. Scale bars = 3 cm. (**b**) The root length after the ethylene treatment of the *ZmSPL12* OE and KO lines and their corresponding controls. (**c**) The stress ratio after ethylene treatment of the *ZmSPL12* OE and KO lines and their corresponding controls. (**d**) The root length of the same lines and controls after treatment with 1 μM ACC. (**e**) The stress ratio of the same lines and controls after treatment with 1 μM ACC. In (**b**–**e**), the values are presented as means ± s.d, with individual data points represented as black circles. Statistical significance was determined using a two-tailed Student’s *t*-test.

It was hypothesized that *ZmSPL12* may function in root penetration through the ethylene signal; *ZmSPL12* OE and KO lines treated with ethylene may exhibit phenotypes similar to those observed under compaction conditions. To confirm this, *ZmSPL12* OE and KO lines were treated with ethylene (Figure 3a, middle) or its precursor ACC (Figure 3a, bottom) for further analysis. After three days of treatment, photographs were taken, and the root lengths of different lines were measured (Figure 3b–e). The statistical results indicated that the root growth of the *ZmSPL12* OE lines was significantly less inhibited by ethylene or ACC treatment compared to the wild-type plants (Figure 3b,d). Conversely, the root growth of the *ZmSPL12* KO lines was markedly more suppressed compared to that observed in the wild-type plants (Figure 3b,d). Stress ratio analysis further confirmed that root growth in the *ZmSPL12* OE lines was less affected by these treatments than in the wild-type, whereas root growth in the KO lines was more affected (Figure 3c,e).

### 2.4. ZmSPL12 Influences Root Elongation and the Development of the Root Cap

**Figure 4:**
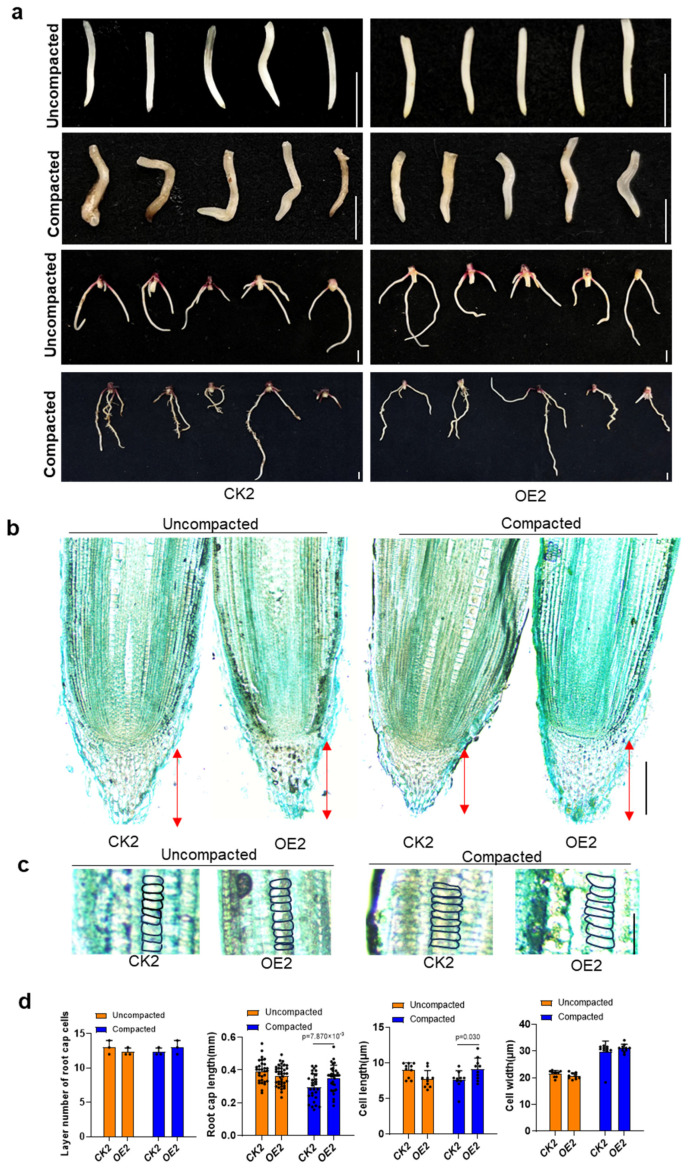
A comparison of the root tip cells between the *ZmSPL12* overexpression lines and controls under non-compacted and compacted soil conditions. (**a**) The changes in primary and crown roots of wild-type and overexpression lines under compacted and uncompacted conditions. Scale bars = 0.5 cm. (**b**) The root cap slices from wild-type and *ZmSPL12* OE lines under uncompacted (**left**) and compacted (**right**) soil conditions. Scale bars = 200 μm. (**c**) Images of cells in the root elongation zones of *ZmSPL12* OE lines and their corresponding controls under uncompacted (**left**) and compacted (**right**) soil conditions. Scale bars = 50 μm. (**d**) Statistics of the root cap length (**left**), cell length (**middle**), and cell width (**right**) of different lines. The values are presented as means ± s.d, with individual data points represented as black circles. Statistical significance was determined using a two-tailed Student’s *t*-test.

To explore the effect of soil compaction on the root tip cells of the *ZmSPL12* OE lines, the root tips of these lines and their wild-type counterparts grown in compacted and non-compacted soils were sampled for paraffin sectioning and staining (Figure 4). The results revealed that soil compaction leads to significantly reduced root cap length in wild-type plants, whereas the *ZmSPL12* OE lines exhibited only minimal change in this trait. Statistical analysis showed that the number of cell layers within the root cap remained comparable between the genotypes regardless of soil compaction, indicating that compaction might primarily inhibit root cap cell elongation. In particular, the *ZmSPL12* OE lines displayed resilience to this compaction effect. Measurements of the root elongation zone showed that soil compaction drastically shortened cell length and broadened cell width in both genotypes. Nevertheless, in the *ZmSPL12* OE lines, the cell length within the elongation zone was relatively unaffected, although there was a slight increase in cell width. These findings were in line with earlier studies, which reported that plants exposed to soil compaction displayed shorter roots and increased root diameters [12,33]. Considering that the *ZmSPL12* OE lines were insensitive to ethylene, their downstream responses remained unchanged, resulting in reduced inhibition of root growth under soil compaction conditions. As a result, the findings suggested that *ZmSPL12* seems to provide a protective mechanism against the detrimental effects of soil compaction on root growth and development.

### 2.5. ZmSPL12 Negatively Regulating Expression Levels of Ethylene-Responsive Genes

**Figure 5:**
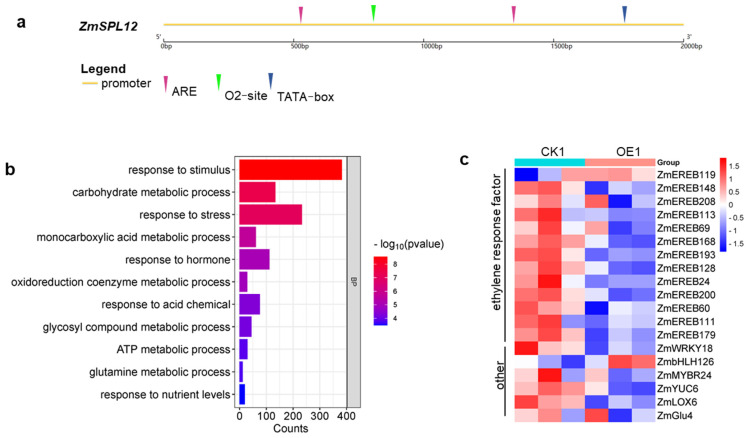
RNA-seq data analysis. (**a**) Promoter analysis of *ZmSPL12*. (**b**) Enrichment analysis of differential genes in *ZmSPL12* overexpression line. (**c**) Heat map of genes related to ethylene in *ZmSPL12* overexpression line.

The qRT-PCR analysis showed that *ZmSPL12* expression level is induced by ethylene treatment (Supplementary Figure S2). Upon analyzing the promoter region of the *ZmSPL12* gene, two essential anaerobic responsive elements (AREs) necessary for anaerobic induction were identified, suggesting a potential connection between *ZmSPL12* expression and hypoxia. To gain deeper insights into *ZmSPL12-*mediated root growth, the roots of *ZmSPL12* OE1 and its corresponding control CK1 were sampled for RNA-seq. GO enrichment analysis of the differentially expressed genes (DEGs) showed that DEGs are mainly involved in stimuli, metabolism, stress, hormones, and other processes (Figure 5).

Subsequently, we examined the expression levels of ethylene-responsive genes between *ZmSPL12* OE1 and its corresponding control, CK1. The results showed that the expression levels of most ethylene-responsive genes in the *ZmSPL12* OE1 were downregulated compared with the wild-type CK1. This finding revealed that the increased expression levels of *ZmSPL12* result in the suppression of transcripts of ethylene-responsive genes in the roots.

EIN/EIL proteins are ethylene-insensitive or ethylene-insensitive-like transcription factors that play a key role in ethylene signaling [34]. In Arabidopsis, mutations in the *ETHYLENE-INSENSITIVE3* (*EIN3*) gene restrict the plant’s response to the gaseous hormone ethylene [35], while its overexpression leads to phenotypes associated with ethylene treatment, indicating that EIL proteins are crucial for the ethylene-mediated regulation of plant development. Studies on rice have demonstrated that the accumulation of ethylene stimulates the activation of *OsEIL1*, which in turn inhibits root epidermal cell elongation and cortical cell expansion by regulating downstream genes. To confirm whether the expression levels of EIL/EIN genes are regulated by *ZmSPL12*, we performed RT-PCR analysis of EIL/EIN genes in the roots of wild-type and *ZmSPL12* overexpression lines under compacted and non-compacted conditions (Supplementary Table S1). The results showed that the *ZmEIN2* expression level was significantly downregulated in the *ZmSPL12* OE line compared to wild-type plants, further supporting that *ZmSPL12* plays a negative role in ethylene signaling, resulting in the reduced inhibition of root growth under compacted soil conditions (Supplementary Figure S3).

To evaluate the potential application of *ZmSPL12* in maize breeding, growth experiments of *ZmSPL12-*overexpressing hybrids in high-density soils were conducted. The results showed that the *ZmSPL12-*overexpressing hybrids have shorter primary roots compared to the corresponding non-transgenic hybrids under normal growth conditions (Supplementary Figure S4). However, when soil density was increased, the primary roots of the non-transgenic hybrids were significantly shortened, while the primary roots of the *ZmSPL12*-overexpressing hybrids were less inhibited and displayed significantly greater root length compared to the non-transgenic hybrids. These results indicated that *ZmSPL12* has great potential in the process of maize breeding improvement.

## 3. Discussion

The root cap plays a multifaceted role in facilitating root penetration into soil, enhancing water and mineral absorption, alleviating heavy metal stress, and modulating the rhizosphere microbiome [36]. The root cap regulates lateral root development and steers growth trajectories through the production of auxin, enabling adaptation to diverse soil conditions [36]. Additionally, the root cap serves to protect the root tip from mechanical harm and senses external stimuli. Its outermost cells are encased in an electron-dense cell wall modification that is analogous to a plant cuticle; targeted degradation of this cuticle can enhance sensitivity to abiotic stressors [37]. In particular, the root cap serves as a pivotal site for ethylene perception [17]. Roots lose their ethylene response upon removal of the root cap, indicating the necessity of a root cap for ethylene sensing. Under compacted soil conditions, the accumulated ethylene was detected at the root cap, which then triggers physiological and biochemical adjustments, leading to decreased root elongation and increased radial expansion [9].

Mucilage secretion and outer cell sloughing aid the root cap in penetrating soil. In loose soil, maize roots with or without caps exhibit comparable growth rates and diameters. However, in compacted soil, the elongation of the root is halved in the absence of a root cap, while the diameter of the roots increases by 30%. This underscores the important role of the root cap in mitigating mechanical resistance, enabling faster growth in dense soils. Longer root caps may further enhance penetration in such conditions [38]. Here, our results showed that *ZmSPL12* plays a positive role in response to soil compaction, with reduced inhibition of the root growth in *ZmSPL12* OE lines (Figure 1 and Figure 2). Moreover, *ZmSPL12* OE lines have longer root caps than wild-type plants in compacted soil conditions, confirming the critical role of the root cap in compacted soil growth (Figure 4).

The AP2-EREBP transcription factor family, encompassing AP2, RAV, DREB, and ERF subfamilies, plays crucial roles in plant growth, hormone responses, and environmental adaptations [39]. These factors are crucial in mediating plant responses to both biotic and abiotic stresses [40,41], including in rice [42], *Arabidopsis* [40], and cotton [43]. Members of the RAV subfamily have also been shown to participate in ethylene responses [44]. Here, the roots of *ZmSPL12* OE lines were insensitive to ethylene or ACC treatments (Figure 3). And the expression levels of ethylene-related genes were reduced in the *ZmSPL12* OE lines, suggesting the *ZmSPL12* function in the ethylene signal pathway rather than ethylene synthesis or transport (Figure 5). The downregulation of these transcription factors conferred insensitivity to ethylene, thereby alleviating the inhibition of root growth under compaction.

Studies in rice have shown that ethylene accumulation activates *OsEIL1*, which boosts auxin biosynthesis via *OsYUC8*, inhibiting epidermal cell elongation and, thus, root elongation [9]. Ethylene signaling also indirectly promotes ABA biosynthesis, contributing to cortical cell radial expansion and further inhibiting root elongation under compaction [9]. Consistent with previous reports, the wild-type maize roots exhibited reduced root elongation and an increase in cell width under compaction. In contrast, the *ZmSPL12* OE lines were insensitive to ethylene, showing minimal cell length changes. This suggests ethylene may regulate cell development via auxin, although the direct regulation of ZmSPL12 by auxin remains uncertain(Figure 4). Our findings indicate that overexpressing *ZmSPL12* influences root elongation under compaction through ethylene, potentially indirectly regulating auxin and ABA, ultimately impacting root tip cell development. Whether *ZmSPL12* directly impacts auxin and ABA levels warrants further exploration.

**Figure 6:**
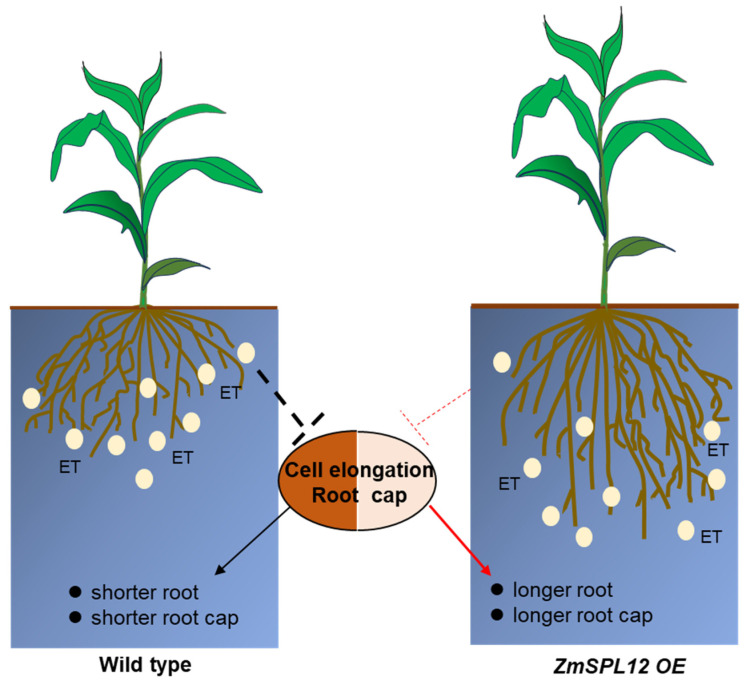
Function mode of *ZmSPL12* under compacted soil. (ET: ethylene).

In this study, we conducted observations and statistical analyses of the compaction phenotype following the overexpression and knockout of *ZmSPL12*, confirming that an increase in *ZmSPL12* expression does indeed influence the plant’s response to soil hardness. Our experiments involving the treatment of ethylene and its precursor ACC further validated that the *ZmSPL12*-overexpressing lines exhibited reduced sensitivity to ethylene (Figure 3). Additionally, root tip slice results obtained under both normal conditions and compaction treatments further supported our findings. Specifically, after soil compaction, ethylene was found to inhibit the growth of root caps and the elongation of cells in the elongation zone. Notably, in the roots of the *ZmSPL12*-overexpressing strain, the inhibitory effect of ethylene on root caps and cell elongation was less pronounced compared to that in the wild type (Figure 6). This observation indicated that the roots of the *ZmSPL12*-overexpressing strain were longer under compaction conditions. Our findings suggest that the overexpression of *ZmSPL12* regulates root growth in compacted soil by modulating the elongation of the root cap and root elongation zone cells. Based on these results, we hypothesize that materials insensitive to ethylene may possess the potential to adapt to compacted soil conditions. Overall, our experiments provide valuable insights and ideas for the cultivation of maize varieties with enhanced root growth capabilities in compacted soil environments.

## 4. Methods

### 4.1. Plant Materials and Growth Conditions

Transgenic maize plants, including OE and KO lines derived from ZC01 (provided by the Life Science Technology Center of China Seed Group Co., Ltd., C2-4 Building, Wuhan National Biological Industry Base, High-tech Development Zone, East Lake, Wuhan), were isolated through self-pollination and PCR analysis. Specifically, the *ZmSPL12* OE lines (OE1 and OE2) and their wild-type counterparts, as well as the *ZmSPL12* KO lines (ko#1 and ko#2) and their corresponding wild-type plants, were subjected to hardness experiments. The greenhouse conditions were carefully controlled, maintaining a temperature of 28 °C, humidity at 30%, and a 12 h light/12 h dark cycle.

### 4.2. Soil Hardness Experiment

The soil was mixed with vermiculite in a 3:1 ratio to achieve a moisture content of approximately 30% and placed in small pots (6 cm × 6 cm × 8 cm). Maize plants were divided into experimental and control groups, with the control group planted in normal soil and the experimental group in compacted soil. Soil hardness was measured using a hardness tester after compacting the soil. After growing for 25 days, the roots were washed, photographed, and then measured.

### 4.3. Soil Bulk Density Experiment

Based on a previous report [10] and our attempts, a bulk density of 0.8 g cm⁻^3^ was considered optimal for maize growth, while 1.2 g cm⁻^3^ represented high soil bulk density. We determined the soil water content (*v*/*w*) to be around 30%. Maize plants were grown in test tubes containing soil of different bulk densities and photographed after 3 to 5 days.

### 4.4. Ethylene Treatment Experiment

Maize seeds that had been germinated on wet paper for three days were subjected to ethylene and ACC treatments. The control group received normal hydroponic treatment, while the experimental group was treated with ACC treatment by transferring the hydroponic maize to a 0.1 μM aqueous solution for three days. For ethylene treatment, maize plants were placed in a sealed box with supports to maintain upright posture. Water was added to maintain humidity, and a specific volume of ethylene was injected into the box. After three days, the root phenotypes were observed and recorded.

### 4.5. Root Tip Sectioning Experiment

Following soil compaction experiments with overexpression lines and wild types, the roots were washed, and the root tip materials were fixed for paraffin sectioning and staining. The sectioning results were then photographed.

### 4.6. RNA Extraction and qRT-PCR Assays

RNA was extracted from maize roots subjected to various treatments, including different regions such as the root tip, elongation zone, root hair zone, maturation zone, and lateral root zone, using a Vazyme Biotech RNA extraction kit. Subsequently, reverse transcription and quantitative reverse transcription polymerase chain reaction (qRT-PCR) were performed to analyze gene expression. Quantitative detection was performed using the Vazyme RNA Quant assay kit, following the protocol. For information on cDNA sequences and sources, please refer to the maizeGDB website (https://www.maizegdb.org/ accessed on 20 August 2024.)

### 4.7. RNA-seq

The *ZmSPL12* OE line and their wild types that were hydroponically cultivated under normal conditions for 15 days had their primary roots collected and sent to a company for sequencing. Each group contained three replicates, with three groups for one line. Subsequently, the data returned by the company were analyzed.

## Data Availability

All data generated or analysed during this study are available within the article or upon request from the corresponding author.

## Supplementary Materials

The following supporting information can be downloaded at: https://www.mdpi.com/article/10.3390/plants13243525/s1, Supplemental Figure S1: Comparison of crown root phenotypes in *ZmSPL12* overexpression and mutant plants under uncompacted and compacted soil conditions, relative to their respective controls; Supplemental Figure S2: Ethylene-induction assay of *ZmSPL12* in different tissues; Supplemental Figure S3: Differential expression of EIL/EIN genes in *ZmSPL12* overexpression lines under non-compacted and compacted conditions; Supplemental Figure S4: Primary root length differences in *ZmSPL12* overexpression hybrid lines under uncompacted and compacted soil conditions relative to their respective controls; Supplementary Table S1: EIL/EIN gene number and primer information.
