# Supplementary material for: ZmSPL12 Enhances Root Penetration and Elongation in Maize Under Compacted Soil Conditions by Responding to Ethylene Signaling
Source: Plants (Basel). 2024 Dec 17;13(24):3525. doi: 10.3390/plants13243525 (PMC11678858; doi:10.3390/plants13243525)
Supplement: Supplementary file 1 [file plants-13-03525-s001.zip › plants-3352488-supplementary.pdf]

Supplementary Material

ZmSPL12 Enhances Root Penetration and Elongation in Maize Under Compacted Soil Conditions by Responding to Ethylene Signaling

Hua Xu [1,2], Zhigang Zheng [3], Lei Ma [1], Qingyun Zhang [1,4], Lian Jin [1,4], Ke Zhang [1], Junjie Zou [1,4,5,*], Hada Wuriyanghan [2,*] and Miaoyun Xu [1,4,5,*]

1 Biotechnology Research Institute, Chinese Academy of Agricultural Sciences, Beijing 100081, China; 15756291121@163.com (H.X.); malei20520@163.com (L.M.); sdwfzqy1996@163.com (Q.Z.); jla13453556961@163.com (L.J.); zhangk4840@163.com (K.Z.)

2 Key Laboratory of Forage and Endemic Crop Biotechnology, Ministry of Education, School of Life Sciences, Inner Mongolia University, Hohhot 010070, China

3 State Key Laboratory for Conservation and Utilization of Subtropical Agro-Bioresources, College of Life Sciences, South China Agricultural University, Guangzhou 510642, China; carlzzg@163.com

4 National Nanfan Research Institute (Sanya), Chinese Academy of Agricultural Sciences, Sanya 572025, China

5 Hainan Seed Industry Laboratory Sanya 572025, China

* Correspondence: zoujunjie@caas.cn (J.Z.); nmhadawu77@imu.edu.cn (H.W.); xumiaoyun@caas.cn (M.X.)

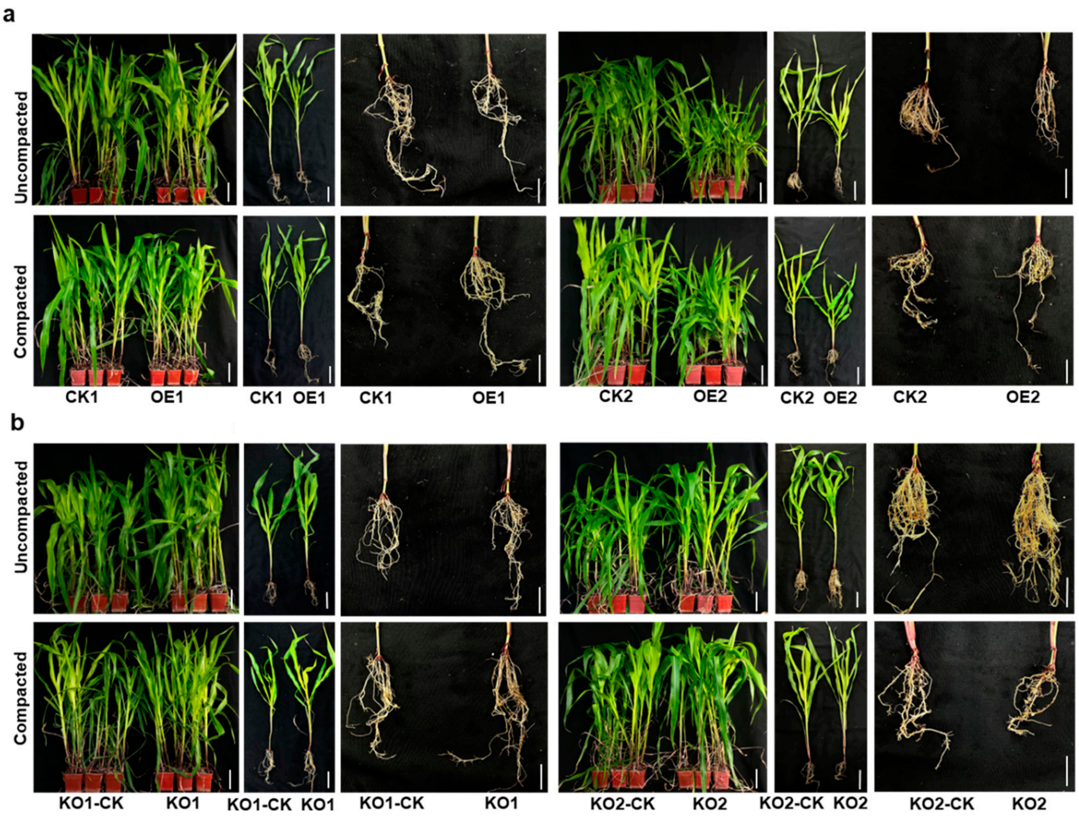

Supplementary Figure S1. Comparison of crown roots phenotypes in *ZmSPL12* overexpression and mutant plants under uncompacted and compacted soil conditions, relative to their respective controls. a. Plants and crown roots phenotypes of the *ZmSPL12* overexpression and the respective controls under uncompacted (top) and compacted (bottom) conditions, scale bars, left and middle, 10cm, right, 5cm. b**.** Plants and crown roots phenotypes of the *ZmSPL12* mutant and the respective controls under uncompacted (top) and compacted (bottom) conditions, scale bars, left and middle, 10cm, right, 5cm.

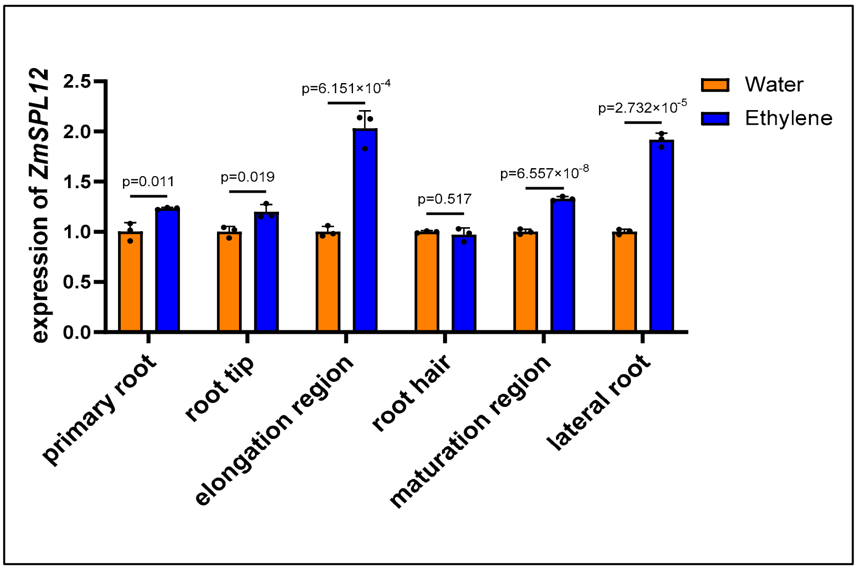

Supplementary Figure S2. *ZmSPL12* was induced by ethylene. Ethylene-induction assay of *ZmSPL12* in different tissues. values are means ± s.d. with individual data points shown as black circles. Statistical analysis was performed using two-tailed Student's *t-test*.

Gene name	Gene number for version V5	Forward primer	Reverse primer
ZmEIL1	Zm00001eb395480	AGTCTGGATTTCAGCCTGCC	CGTCCATCTGGATCCTCTGC
ZmEIN2	Zm00001eb119690	GGCACTGTCACAGGGAAGAA	TCTGCAAGTACCATAGTCAGC
ZmEIL3	Zm00001eb015440	AAGGAGATCTCGACCTGGCT	TCCCCCTTGCACTCATCAAC
ZmEIL4	Zm00001eb316730	GGCGAATGTGGAAGGACAGA	ACCTGCATGAGCTTGAGCA
ZmEIL13	Zm00001eb034980	GGTGTTCTACAACGCGAGGA	GCGGTCGACATCATACTCGT

Supplementary Table S1. EIL/EIN gene number and primer information.

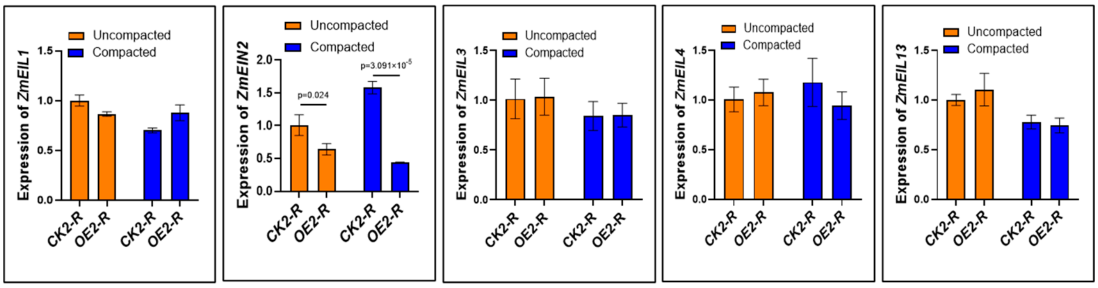

Supplementary Figure S3. Differential expression of EIL/EIN genes in *ZmSPL12* overexpression lines under non compacted and compacted conditions.

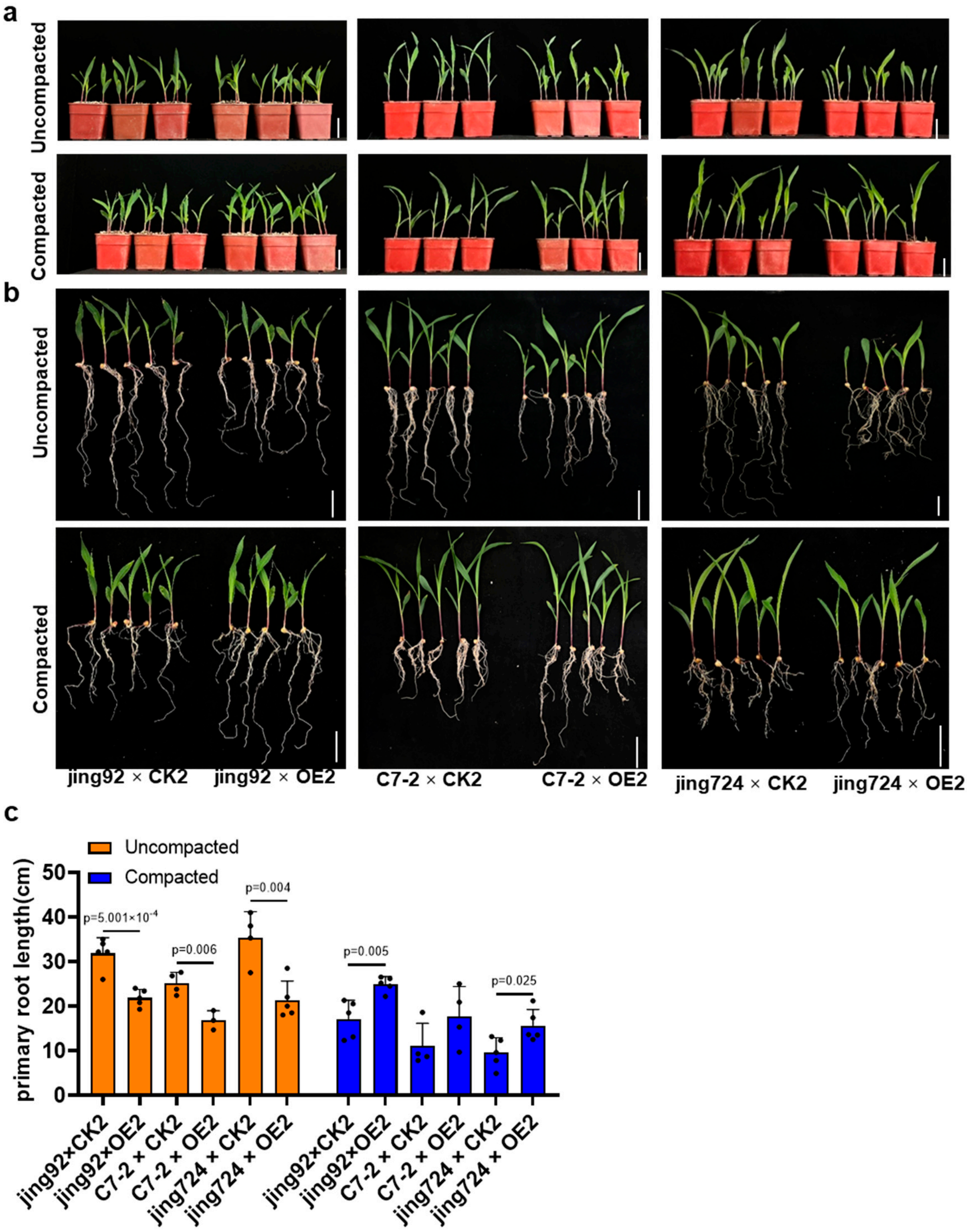

Supplementary Figure S4. Primary root length differences in *ZmSPL12* overexpression hybrid lines under uncompacted and compacted soil conditions relative to their respective controls. a-b. Seedlings and primary roots phenotypes of the *ZmSPL12* hybrids and the respective controls under uncompacted (top) and compacted (bottom) conditions, scale bars, 5cm. c. Primary root length of the *ZmSPL12* hybrids and the respective controls under uncompacted and compacted conditions, values are means ± s.d. with individual data points shown as black circles. Statistical analysis was performed using two-tailed Student's *t-test*.
